# The Role of Thyroid Hormone Signaling in the Development and Pathophysiology of Hearing: From Molecular Mechanisms to Clinical Applications

**DOI:** 10.3390/ijms27052196

**Published:** 2026-02-26

**Authors:** Yuan Jin, Shang Li, Qiong Zhang, Bei Guo, Ying Xiong, Yao Hu, Haixia He, Pei Gao, Wei Chen

**Affiliations:** Department of Otorhinolaryngology, The Central Hospital of Wuhan, Tongji Medical College, Huazhong University of Science and Technology, Wuhan 430014, China; m201975754@alumni.hust.edu.cn (Y.J.); m202476567@hust.edu.cn (S.L.); zq54zq@126.com (Q.Z.); guobei626@163.com (B.G.); free_yingying@foxmail.com (Y.X.); huyao125@sina.com (Y.H.); enthehaixia@163.com (H.H.); asteria0206@163.com (P.G.)

**Keywords:** thyroid hormone, hearing loss, Cochlear development, hair cell, auditory

## Abstract

Hearing loss (HL) is the most common sensory disorder, affecting over 430 million individuals, and its prevalence continues to rise steadily. Thyroid hormone (TH) signaling is a key endocrine regulator that critically governs key processes in cochlear development, such as sensory hair cell differentiation, ion channel expression, and synaptic maturation. TH deficiency can lead to different types of hearing loss, with or without cochlear structural deformity. Moreover, TH deficiency in pregnant women can cause hearing impairment in newborns. This review synthesizes the mechanisms by which TH signaling contributes to cochlear development and pathogenesis of hearing loss. By integrating insights from human studies and animal models, we discuss the prognostic significance and clinical applicability of TH signaling levels, highlighting the indispensable role of TH signaling in advancing personalized strategies for the diagnosis and management of HL.

## 1. Introduction

More than 5% of the world’s population (430 million people) suffer from disabling hearing loss, and by 2050, this proportion will even exceed 10% [1]. At present, the recognized etiologies include genetic defects, infections, ototoxic drugs, endocrine disorders, cochlear developmental malformations, trauma, and noise [2,3,4]. Different etiologies lead to different influences on the occurrence and outcome of HL [5,6]. Thyroid Hormone (TH) Signaling Deficiency is a globally prevalent endocrine disorder [7]. Both abnormal changes in serum thyroid hormone levels and genetic disorders affecting thyroid development, hormone biosynthesis, and signaling can lead to sensorineural and conductive hearing loss [8,9]. A high incidence rate of hearing loss was observed in children and adults with endemic iodine deficiency disorders, hypothyroidism, and thyroid hormone resistance [10,11,12,13,14]. A cross-sectional study also indicated the association between thyroid diseases and sudden sensorineural hearing loss [15].

TH has been shown to play an essential role in auditory processes, regulating processes from cochlear patterning and hair cell differentiation to ion channel expression, synaptic maturation, and even the development of the ossicles [16,17,18,19]. Rodent models of congenital hypothyroidism exhibit significant congenital deafness with cochlear structural developmental abnormalities, such as partial loss of hair cells, malfunction of the mechanosensory hair cells, defects in the organ of Corti, and deformities of the tectorial membrane, while exogenous TH administration rescues hearing loss caused by congenital hypothyroidism [20,21,22,23,24]. Interestingly, supplementation with T3 can also accelerate the development of the cochlear inner sulcus and the tunnel of Corti (TC) [25]. These increasing pieces of evidence suggest a strong link between TH signaling and cochlear development as well as the maintenance of auditory function.

Based on the irreplaceable role of TH in embryonic development, developed countries have gradually incorporated TH levels testing into newborn screening. Recent clinical studies have suggested an association between thyroid function and SSNHL. For instance, in patients with moderately severe-to-profound SSNHL, lower levels of FT3 and TSH have been identified as independent risk factors for its occurrence, while a post-treatment rise in TSH levels was shown to be an independent predictor of hearing recovery [13]. However, for children and adults with hearing loss, TH levels testing has not yet been used as a routine etiological diagnostic test, which may lead to missing the treatment window period.

In this article, we review the physiological and pathological roles of TH signaling in cochlear and hearing development. The purpose of this review is to discuss the prognostic significance and clinical applicability of TH signaling levels, which is valuable in advancing personalized strategies for the diagnosis and management of HL.

## 2. Local TH Signaling Levels and Their Regulatory Mechanisms in the Cochlea

**Figure 1:**
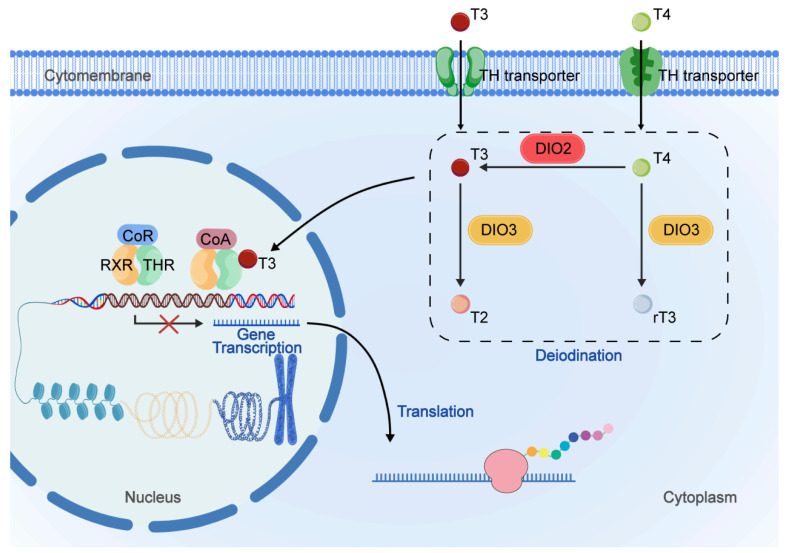
Regulatory mechanisms of the TH signal during cochlear development in mice. Intracellular transport of TH relies on TH transporters located on the cell membrane. DIO2 converts T4 to bioactive T3 via deiodination. DIO3 inactivates TH by converting T4 to rT3 and T3 to T2. In the absence of T3, co-repressors bind to thyroid hormone response elements (TREs) occupied by retinoid X receptor (RXR)-thyroid hormone receptor (THR) heterodimers, thereby actively repressing target gene transcription. Upon T3 binding to the RXR-THR dimer, co-repressors dissociate, and co-activators are recruited, ultimately activating gene expression. Although T4 may also trigger limited transcriptional regulation, T3 is considered the main active form of TH.

In cochlear cells, the concentrations of bioavailable T4 and T3 are regulated by both systemic factors (serum levels) and cochlear local mechanisms (involving deiodinases). Type 2 deiodinase (DIO2) and type 3 deiodinase (DIO3) are the main deiodinases expressed in the cochlea [20]. DIO2 catalyzes the removal of a 5′-iodine from circulating T4 to generate T3, which serves as the primary source of free T3 in the cochlea and other target tissues (Figure 1). Conversely, DIO3 converts T4 to reverse T3 (rT3) and T3 to 3,3′-diiodothyronine (T2); neither metabolite can activate the thyroid hormone receptor (TR). In the cochlea at postnatal day 10 (P10), Dio2 expression is predominantly localized to the bony capsule and fibrocyte regions of the spiral limbus and spiral ligament [21,22]. However, minimal DIO2 expression was detected in the organ of Corti and adjacent epithelium, and not in root cells or tympanic border cells. In contrast, low levels of DIO2 expression have been found throughout the whole cochlea of adult mice. In the cochlea of neonatal mice, DIO2 activity rose dramatically from postnatal day 2 (P2) until it reached a peak at P7, while the activity of DIO2 reduced sharply during P8 to P12 and persisted at low levels into adulthood. DIO2 mRNA expression also increased from birth to a peak at P7 and showed a decreasing trend after P8 [21,22]. In contrast, DIO3 activity decreased postnatally and remained at low levels into adulthood [23]. Publicly available RNA sequencing and microarray datasets also showed a sharp peak of DIO2 mRNA expression level from E16 to P7 (Supplementary Figure S1A). The differential postnatal expression and activity of DIO2 and DIO3 in the neonatal mouse cochlea suggest a trend of T3 signal amplification during cochlear development. Meanwhile, circulating levels of T3 and T4 during the postnatal period also increased between P5 and P16 [24]. In unison, concentrations of T3 and T4 increased in cochlear extracts. These remarkable changes in DIO2 and DIO3 expression and TH content (both in serum and cochlea) indicate the amplification of local T3 levels in the postnatal phase of cochlear maturation before the onset of hearing.

Circulating thyroid hormone levels do not fully reflect the TH concentrations available to local cells. Intracellular uptake of TH is mediated by transporter proteins located at the plasma membrane (Figure 1). TH transporters Oatp1c1 (Slco1c1) mRNA expression levels increased sharply from E18 to P15 in cochlea, while Lat1 (Slc7a5), Mct8 (Slc16a2), and Mct10 (Slc16a10) moderately increased from E18 to P5 [26]. Publicly available RNA sequencing and microarray datasets also showed an increasing trend in the expression levels of TH transporters (Slc16a2, Slco1c1, Slc10a1, Slco2b1, Slc7a5) in the developmental cochlea (Supplementary Figure S1B–D). Increased mRNA expression of TH transporters suggests increased intracellular bioavailability of TH in development cochlea of neonatal mice.

Intracellular TH binds to nuclear receptors and therefore acts on downstream gene transcription (Figure 1). THRα1 (encoded by *THRA*), THRβ1, and THRβ2 (encoded by *THRB*) are the main effective TH receptors (THR) in the cochlea. In the P4 immature cochlea, *Thrb* mRNA could be detected, mainly expressed in the greater epithelial ridge (which includes columnar epithelial cells located medial to inner hair cells) [27]. In other P4 cochlear structures, low THRβ mRNA expression was also detected, such as the lesser epithelial ridge, Hensen’s cells, stria vascularis, and the vestibular membrane. In neonatal mice, *Thrb* mRNA expression remained relatively constant during P3 to P7 but decreased during P7 to P15 [26,28]. Publicly available RNA sequencing datasets also showed a relatively constant but gently increasing mRNA expression level of *Thrb* from E16 to P7 (Supplementary Figure S1E). The *Thra* mRNA isoform is highly expressed in the spiral ganglion and expressed relatively lower than *Thrb* in the greater epithelial ridge, the sensory epithelium, and the lateral wall of the cochlear. Although no significant changes in THR expression level were observed in the developing cochlea, the differential expression of THR isoforms suggested the cochlear cell specificity of TH signaling. THRα1-deficient mice have a normal auditory-evoked brainstem response, while THRβ-deficient mice show severe hearing loss, and this suggests the differential regulation between *THRA* and *THRB* in cochlear function and morphological development [29]. THRα/THRβ double knockout mice have a more severe hearing phenotype. Overexpression of THRα could improve hearing defects caused by THRβ knockout, suggesting THRα and THRβ may have merely redundant effects in the cochlea.

**Figure 2:**
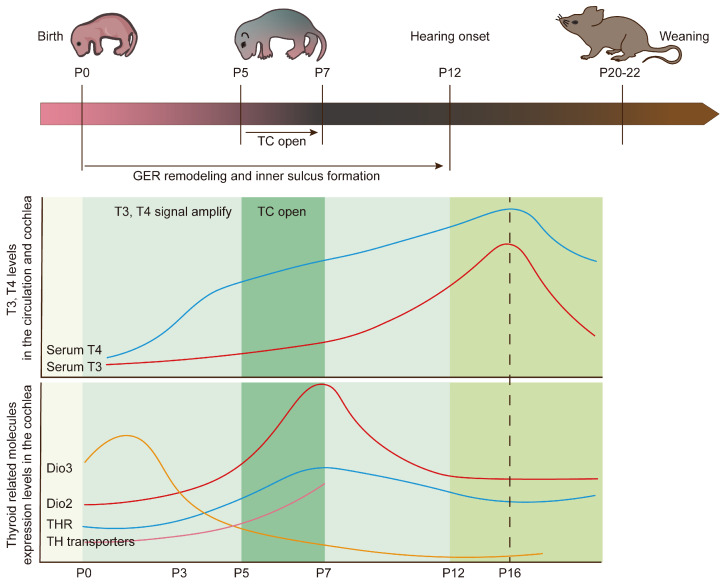
Cochlear structural remodeling in mice is synchronized with postnatal amplification of TH signaling. The opening of the TC, the remodeling of the greater epithelial ridge (GER, also known as Kölliker’s organ), and the formation of the inner sulcus are critical structural changes that occur during postnatal cochlear development. In neonatal mice, serum T3 and T4 increase from postnatal day 0 (P0), peak around P16, and gradually decline thereafter. DIO2 mRNA expression rises postnatally to a peak around P7 and then declines after P8, whereas DIO3 mRNA expression shows a rapid decline during the same period. Postnatal upregulation of TH receptors (THRs) and TH transporters also contributes to the amplification of local TH signaling. These key cochlear remodeling events are thus closely associated with the temporal dynamics of TH signaling in the developing cochlea.

In summary, the elevation of TH levels in the circulation and the high expression of deiodinases, THR, and TH transporters in the cochlea during the first postnatal week synergistically promote local TH signal amplification (Figure 2).

### 2.1. TH Signaling Level Disorder Results in Hearing Impairment

The relationship between thyroid dysfunction and hearing loss was recognized over 100 years ago. Areas having a high prevalence of iodine deficiency-induced endemic goiter also show increased prevalence of all forms of congenital deafness [8]. A large number of case reports and cohort studies have also shown the correlation between thyroid dysfunction (maternal, central, or glandular hypothyroidism) and various forms of hearing loss (congenital, progressive, sudden, conductive, sensorineural, or mixed) [9,30,31,32,33,34,35]. Hearing impairment associated with congenital or acquired hypothyroidism is most often bilateral, symmetrical, and sensorineural, with some cases also having conductive or mixed loss. Due to the heterogeneous hypothyroid status of patients with hypothyroidism, the degree of HL may range from asymptomatic to mild to profound. After thyroid hormone replacement therapy, the degree of HL could be reversed, while some studies have also shown that CH patients have persistent hearing loss, suggesting structural damage to the cochlea or central nervous system caused by TH signal deficiency. Drug-induced TH deficiency between the late embryonic stage and the onset of hearing in altricial rodents also showed that congenital hypothyroidism can cause severe hearing loss [14,36,37,38,39,40]. Disruption of the local TH signal or resistance to TH in the cochlea caused by abnormal expression of deiodinase, THR, and TH transporter can also affect auditory and cochlear structural maturation [41,42,43,44]. *Thra* mutation, including both TRα2 and TRα1 deletion, was reported not to cause hearing loss, suggesting that *Thra* is mainly involved in the regulation of cochlear structural development [45,46]. In contrast, *Thrb* deficiency leads to deafness, demonstrating the necessity of *Thrb* in regulating auditory function. Although there are distinct physiological roles of *Thra* and *Thrb*, altering the expression levels of THRα1 and THRα2 can compensate for the loss of Thrb. Thyroxine replacement treatment can partially rescue TH deficiency-induced hearing loss [47,48]. It should be emphasized that not all patients receiving thyroid hormone treatment can improve their hearing [30]. Moreover, hyperthyroidism is associated with susceptibility to hearing loss [49,50] or tinnitus [51]. Previous reports also associated hearing loss with Pendred syndrome, caused by biallelic mutations in the *SLC26A4*/*PDS* gene, and characterized by the combination of sensorineural deafness and goiter. The thyroid function of these Pendred syndrome patients could be hyperthyroidism, euthyroid, or hypothyroid [52,53,54,55,56]. Neonatal mice treated with T3 in the early postnatal period also exhibit severe hearing loss [49,57]. In contrast, hearing loss is commonly related to the use of antithyroid Thionamides or Teprotumumab during patients’ acceptance of hyperthyroidism (especially Graves’ disease) treatment [49,58,59,60]. TH signaling defects in children and adults also may lead to sudden sensorineural hearing loss. The mechanism by which low T3 levels lead to SSNHL and ear stuffiness is speculated to be related to vascular and hemodynamic changes caused by low tissue metabolism [61]. Low FT3 and TSH levels have been identified as risk factors for moderate to severe SSNHL [13]. Due to the positive correlation between elevated TSH and hearing recovery in patients with moderate to severe SSNHL, the TSH level could be considered as an independent outcome predictor [13].

Considering the potential benefits of early use of alternative therapy for preserving cochlear structure and hearing, it is reasonable for refractory SSNHL patients with low TH levels or high TSH levels to supplement thyroxine as early as possible. In fact, hearing impairment has been identified as having a significant impact on the development of speech, psychological well-being, educational skills, and social status in children and adolescents. It is also associated with an increase in cognitive impairment and dementia in the elderly. For both congenital and sudden hearing loss related to TH deficiency, providing treatment during critical window periods can help minimize language delay or disease progression caused by a lack of exposure to sound. Therefore, the advancement of screening leading to earlier detection is very necessary.

### 2.2. TH Signal Regulates Cochlear Structure Development

**Figure 3:**
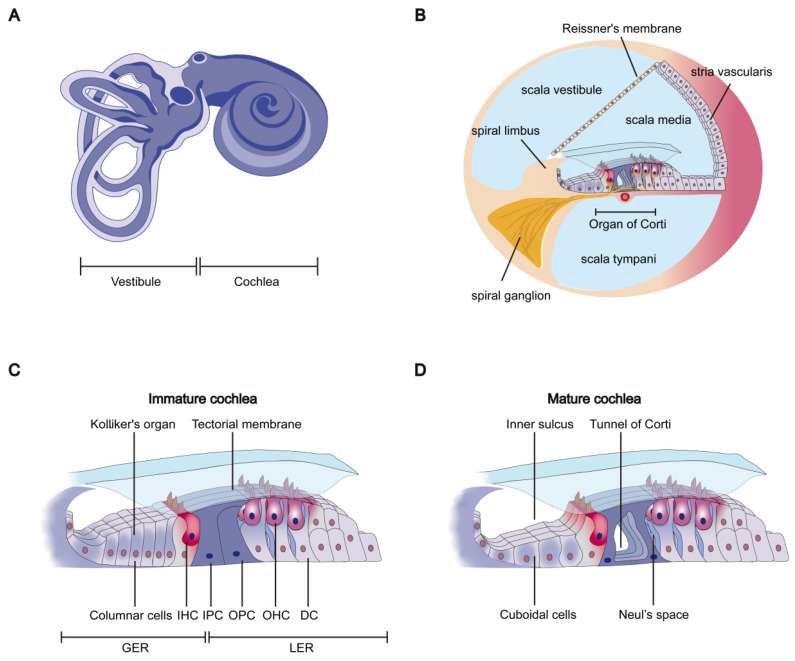
Postnatal morphological remodeling of the cochlea. The inner ear of mammals consists of the cochlea, the vestibule, and the semicircular canals (**A**). The cochlea is responsible for perceiving sound, while the vestibule and semicircular canal system are responsible for maintaining balance. In the cochlea, there is a series of interconnected soft tissue sacs and ducts known as the membranous labyrinth, which consists of three ducts: the scala vestibuli, scala media, and scala tympani (**B**). The scala media (cochlear duct) is a coiled tube filled with endolymph, with three unique walls including Reissner’s membrane, the stria vascularis, and the basilar membrane. The sensory epithelium (organ of Corti, OC) comprises two types of mechanosensory hair cells (IHCs and OHCs), located on the basilar membrane. Two non-sensory cell regions, the greater epithelial ridge (GER) and the lesser epithelial ridge (LER), flank the OC. The GER, composed of columnar cells, as well as the closely adjacent IPCs and OPCs, is characteristic of an immature cochlea (**C**). The most significant morphological remodeling of the cochlea during postnatal development is the degeneration of GER cells and the separation of pillar cells, resulting in the formation of the inner sulcus and the TC in the mature cochlea (**D**).

The mammalian inner ear is derived from the otic placode, which arises as a paired dorsolateral ectodermal thickening in the developing embryo [62,63,64]. The critical period of inner ear development and hearing maturity in mice corresponds approximately to the time interval from the early embryonic stage to postnatal day 18, whereas in humans, it extends from the embryonic stage to the first year after birth. The mammalian cochlea finally develops a spiral structure that consists of adjacent membranous canals and is surrounded by a bony shell, forming a snail shell-like morphology (Figure 3A,B) [65]. The cochlea acts as the auditory sensory organ, sensing mechanical stimuli caused by sound and vibration [17,18,66,67]. The morphogenesis and development of the cochlear duct postnatally continue in neonatal mice. The most significant cochlear structural remodeling is the degeneration of GER and separation of pillar cells TC, leading to the formation of the inner sulcus and the TC (Figure 3C,D) [68,69]. Different cochlear structures such as the stria vascularis (SV), GER, and OC may exhibit varying sensitivities to TH, despite all of these structures being immersed in the endolymph, and the period of maximal sensitivity of these structures to TH almost all coincide with the developmental stage during which these structures undergo major morphological changes (P6–P14 for the GER or inner sulcus [70]; the first postnatal week for the tectorial membrane; P5–P10 for pillar cells and TC; and the second to third postnatal weeks for OHC synaptogenesis) [39].

#### 2.2.1. Hair Cell

Auditory hair cells (HCs) can sense the sound vibration transmitted into the cochlea and convert such mechanical vibrations into electrical signals, which are then transmitted to the auditory center of the brain via the auditory pathway. There is a single row of inner hair cells (IHCs) in the cochlea near the modiolus, and three rows of outer hair cells (OHCs) located on the SV side. Mechano-electrical transduction (MET) occurs in both IHCs and OHCs. OHCs provide amplification/active mechanics, while IHCs primarily drive auditory-nerve signaling [17,18,71].

TH plays a crucial role in the differentiation, function, maturation, and survival of HCs. Hypothyroidism caused by methimazole results in morphological defects in sensory OHCs, impairing the accumulation of actin, as well as stiff OHCs and pillar cells (PCs) [72]. The electromotility and potassium current development of HCs depend on TH regulation [73,74]. Sendin G et al. have reported that IHCs of 2-week-old athyroid Pax8 deficiency mice lacked large-conductance Ca^2+^-activated K^+^ channels and KCNQ4 channels [75]. Mice with hypothyroidism at P14–P16 were detected to lack rapid but graded membrane potential responses to sensory input and efficient Ca^2+^ influx–exocytosis coupling, which may be related to the loss of transcriptional regulation functions of TH [75]. Brandt N et al. have also demonstrated the abnormal postnatal spiking activity and expression of Ca^2+^ and K^+^ channels in IHCs of a TH deficiency rodent model [76]. In addition, fast-activating potassium conductance *I_K,f_* in IHCs was largely absent at P15–P18 in THRβ-deficient mice, while *I_K,f_* was clearly expressed at P18 in WT mice of the same genetic background. On the contrary, THRα1-deficient mice displayed *I_K,f_* in accord with normal hearing [74]. Moreover, hypothyroidism in rodents causes loss of HCs [77,78]. Mirna M et al. have reported significant OHC loss and permanent reduction in KCNQ4 (the voltage-dependent K^+^ channel) expression and current levels in OHCs of Pit1^dw^ mutant mice, which lack distortion product otoacoustic emissions and cochlear microphonic responses, suggesting defects in OHC function [79]. OHC degeneration was also observed in the Pax8 deficiency model [80].

TH affects the function and structure of hair cells through transcriptional regulation. T3 has been demonstrated as the primary transcription factor for prestin, the molecular motor of hair cells, and a direct or indirect regulator of subcellular prestin distribution. Hypothyroidism not only leads to a significant reduction in the expression of prestin mRNA and prestin protein but also causes alterations in the distribution of prestin in outer hair cells. In Pit1^dw^ mutant mice, prestin expression exhibits a developmental delay in OHCs [79]. Cochlear TH levels can also regulate Fgf-receptor expression. In hypothyroidism conditions, a delay in the down-regulation of Fgfr3 expression occurs in OHCs, which leads to a delay in the development of the inner ear [81].

Early postnatal injection of T3 causes a disorganized arrangement of OHC hair bundles and impaired function of mechanoelectrical transduction of OHCs in WT mice, accompanied by severe hearing loss. In contrast, injection with T3 at a later postnatal stage (after P3) does not cause significant damage to hearing and fiber bundles [25]. These findings indicate that inner ear T3 plays a critical role in the normal differentiation and maturation of inner ear hair cells, and this regulatory effect depends on the action of T3 at an appropriate concentration within the correct time window [20].

#### 2.2.2. Ribbon Synapses

Ribbon synapses form connections between HCs and spiral ganglion neurons (SGNs) and function in the neural transmission process. In neonatal rodents, ribbon synapses continue to develop and mature during the postnatal stages, including synaptic pruning, migration, and refinement. Hypothyroidism caused by Pax8 deficiency results in smaller, abnormal Ca^2+^ currents being observed in IHCs during P6-P8, with abnormal, elevated Ca^2+^ currents at the end of the second postnatal week, and low efficiency of Ca^2+^ influx in triggering exocytosis of the readily releasable vesicle pool in IHCs. Though ribbon synapses could finally be formed in Pax8-deficient mice, the immature ultrastructure of these synapses was observed. Moreover, immature pre-synaptic and synaptic punta, impaired ribbon diffusion, and delayed ribbon migration were independently observed by Yu C et al. in IHCs and OHCs of the Pax8-deficient mice, causing morphological maturation and impairing the pruning of synaptic ribbons [82]. Hyperthyroidism caused by an additional T3 supplement could promote synaptic pruning in wild-type (WT) mice after P4, while the auditory functions were preserved [83]. TH substitution could restore the ribbon synapse morphology and function [83].

#### 2.2.3. Spiral Ganglion Neurons

The IHCs and OHCs in the mature cochlea are innervated by the SGNs. Mature IHCs are innervated mono-synaptically by 5–30 type I SGN fibers, whereas each type I SGN receives input from only one IHC. Type II SGNs constitute 5–10% of all SGNs and contact multiple OHCs. The neonatal mouse cochlea contains an overabundance of type II SGNs that form synapses with OHCs before the first postnatal week; these excess type II SGNs subsequently degenerate, and superfluous afferent synapses are pruned back by the onset of hearing. However, hypothyroidism has been shown to cause abnormal persistence of afferent dendrites and incomplete development of efferent terminals. Rueda et al. have shown that hypothyroidism affects the maturation and differentiation of SGNs. In rats with hypothyroidism induced by PTU administration via gastric tube, type II SGN cell death was not observed, and there was no differentiation into type I and type II neurons [84]. Sundaresan et al. reported a defect in the pruning of afferent type II spiral ganglion neurons in the cochlea of hypothyroid mice generated by continuous feeding of pregnant and newborn mice with a PTU-containing diet, as well as a delay in efferent attachment and maturation of otoferlin expression [85].

#### 2.2.4. Tectorial Membrane

The tectorial membrane (TM) is a ribbon-like strip of highly hydrated extracellular matrix that attaches along its medial side to the surface of the spiral limbus and overlaps HCs’ hair bundles. TM comprises water (97%), glycosaminoglycans, collagenous, and noncollagenous proteins (α-tectorin, β-tectorin, etc.). TM’s movement is not only a response to sound-induced vibrations of the basilar membrane driving the hair bundles, but also propagates traveling waves at audio frequencies along its length. The wide deformed TM continuously lying on KO could be observed in different TH-deficient rodent models [79,86,87,88,89,90,91]. TM in Pit1^dw^ mutant mice showed a clearly abnormal protrusion, with increased expression of both α-tectorins and β-tectorins [79]. The TM in the cochlea of drug-induced hypothyroidism rats also showed a hump-shaped appearance, while cochlear β-tectorins were significantly reduced [92]. In Dio2-deficient mice, the striated sheet matrix was disorganized in the middle and upper regions of the malformed TM, with no disturbing expression of α- and β-tectorin glycoproteins in TM [24]. Mice with a homozygous Slc26a4 mutation were characterized as having a thicker tectorial membrane with reduced β-tectorin protein expression [92].

#### 2.2.5. Kölliker’s Organ and Inner Sulcus

Kölliker’s organ (KO), also referred to as the greater epithelial ridge (GER), is composed of a dense array of columnar epithelial cells located from the spiral limbus to the inner hair cells [93]. KO supporting cells can release adenosine triphosphate (ATP) to initiate a cascade of intracellular Ca^2+^ signaling in adjacent IHCs through connexin hemichannels, which promotes auditory neuron maturation and synaptic refinement before hearing [94]. KO was confirmed as a transient structure that finally remodels into the inner sulcus due to columnar cells being replaced with cuboidal cells during postnatal development [94]. This remodeling of KO may be related to programmed apoptosis of columnar cells. In rodents with hypothyroidism induced by PTU, thick KO and columnar cells persist for a prolonged period and do not transform to cuboidal cells, with no shaped inner sulcus observed in the cochlea [87]. Excessive T3 can cause premature KO remodeling, apoptosis in the GER, and formation of the inner sulcus in WT mice, by mediating T3-stimulated cell apoptosis [25].

#### 2.2.6. Stria Vascularis

Scala media is full of cochlear endolymph, an extracellular solution containing 150 mM K^+^, and thus exhibits a positive potential of +80 mV [95]. When the basilar-membrane motion is elicited by acoustic stimuli, the hair bundle of HCs is exposed to endolymph, and the stereocilia on the hair bundle are deflected so that the mechanoelectrical transduction channel opens. This process permits endolymphatic K^+^ to enter hair cells, resulting in their electrical excitation. The EP accelerates the entry of K^+^ from the ion channel into HCs, causing HC electrical excitation. A lack of EP in the mouse cochlea causes hearing loss, suggesting EP is essential for proper hearing [96,97]. The formation of endolymph potential (EP) is the result of multiple ion transport via the stria vascularis (SV) [97].

In Pit1^dw^ mutant mice, abnormal accumulation of dark deposits was observed in the stria vascularis, accompanied by a smaller width of the stria [79]. Permanently reduced expression of KCNJ10 in the specific stria vascularis region, along with substantially reduced EP, was also observed in the Pit1^dw^ mutant mice, which may contribute to hearing loss [79]. In THRβ-deficient mice, normal EP was formed, although TH signal can regulate Na, K-ATPase, which suggests that defective *I_K,f_* expression, and hearing loss in TRβ-deficient mice do not result from the EP reduction [74].

#### 2.2.7. Pillar Cell and Tunnel of Corti

During cochlear development, the lateral membranes in the middle of IPCs and OPCs separate, forming a triangular space filled with perilymph between adjacent PCs, which is the TC. A delay in the opening of the TC was observed in different hypothyroidism rodent models, including the Pit1^dw^ mutant, pituitary TSH-deficient *Cga* mutants, and pharmacologically induced hypothyroidism [39,45,74,79]. Emphatically, no TC structure in Pit1^dw^ mice could be observed at P12, but the morphology of TC was indistinguishable from that of WT mice at P21, suggesting that the development of TC caused by hypothyroidism is a temporary delay rather than a permanent termination. A similar delay in TC development is also observed in mice with disrupted TH receptors. Although the factors that mediate the development of the TC opening remain unknown, disruption of microtubule formation and decreases in acetylated tubulin in the PCs in hypothyroid rodents may be the reason for the delay of TC opening [72,81,98]. This cytoskeleton developmental disorder in PCs is also a hallmark pathological change in Cx26 deficiency mice, which also have delayed TC opening and classic congenital deafness [99,100,101]. The delay of TC opening and the malformation of OC are considered the main deafness mechanisms of Cx26 deficiency [101]. More interestingly, Dettling et al. have demonstrated that the deafness upon deletion of THRβ function originates outside of hair cell defects [102]. These subtle correlations demonstrate the importance of TC structure in hearing.

### 2.3. Human Temporal Bone Findings in Acquired Hypothyroidism

Parving et al. examined temporal bones from an 83-year-old woman with myxedema and found no morphological alterations or glycosaminoglycan deposition; the observed high-frequency hearing loss was judged consistent with presbycusis [102]. The same group evaluated hearing in 15 myxedematous patients (median age 48 years) before and after levothyroxine treatment and found no improvement in hearing sensitivity, nor was the degree of hearing loss greater than that of age-matched controls. The authors concluded that no causal association exists between adult-onset myxedema and hearing impairment [102]. Similarly, Hald et al. reported histological findings from four temporal bones of patients who died with untreated myxedema (age range 67–95 years). Despite biochemically confirmed severe hypothyroidism, no deposition of acid glycosaminoglycans—a hallmark of myxedema in other connective tissues—was detected in the cochlea. Spiral ganglion cell loss observed in two cases was attributed to advanced age rather than thyroid deficiency [103]. Collectively, the temporal bone specimens from older adults with acquired hypothyroidism did not exhibit marked structural changes attributable to thyroid hormone deficiency.

## 3. Discussion

The cochlea is a structurally complex sensory organ. Distinct cell types are arranged in a rigorous cellular mosaic and extend along the length of the cochlear spiral [62]. The assembly of this structure requires a complex and high-precision regulation network. TH signaling serves as a master regulator of cochlear development, orchestrating a precisely timed sequence of structural and functional maturation events [20]. This review synthesizes current evidence demonstrating that TH exerts its effects through a tightly coordinated network of deiodinases (DIO2, DIO3) [22,23], TH transporters (MCT8, OATP1C1, LAT1) [78], and nuclear receptors (THRα, THRβ) [28,46,74], all of which exhibit spatiotemporally regulated expression patterns in the developing cochlea. The postnatal surge in circulating TH levels, coupled with local amplification of T3 via DIO2 and increased expression of TH transporters and receptors, creates a critical window during which TH drives essential remodeling processes, including hair cell differentiation, ion channel expression, ribbon synapse maturation, pillar cell separation, tectorial membrane organization, and degeneration of Kölliker’s organ. This intricate regulation underscores the critical importance of spatiotemporally precise TH signaling as a master coordinator of cochlear development, ensuring the final maturation of diverse cellular components essential for auditory function. Consequently, the structural integrity and functional maturation of the cochlea, from hair cell differentiation and synapse refinement to the formation of specialized microenvironments like the tunnel of Corti and endolymph composition, are exquisitely dependent on optimal TH signaling during specific postnatal windows. Disruption of any component of this intricate network—whether due to iodine deficiency, congenital hypothyroidism, genetic mutations in TH signaling pathway genes, or even maternal TH insufficiency—can result in permanent hearing impairment. Importantly, the severity, reversibility, and phenotypic presentation of TH-related hearing loss may depend on the timing, duration, and nature of the insult, as well as the specific cellular targets affected.

Meanwhile, the strict developmental dependence of cochlear maturation on TH highlights the limited therapeutic window for intervention in congenital hypothyroidism to prevent permanent auditory deficits, emphasizing the need for early diagnosis and timely hormone replacement. This temporal constraint is strongly supported by clinical evidence from children with congenital hypothyroidism (CH). Rovet et al. prospectively studied a cohort of 101 children with CH identified by newborn screening and found that 20% (15 cases) exhibited mild hearing impairment despite early treatment [33]. Crucially, children with hearing loss began levothyroxine therapy significantly later than those with normal hearing (mean 22.3 vs. 13.5 days of age), whereas disease severity and treatment dose did not differ between groups. Rovet et al. point out that the third week of life represents a critical window for thyroid hormone-dependent cochlear maturation, and that even a modest delay in treatment initiation—from the second to the third week—confers an increased risk of permanent auditory deficits [33]. These findings underscore that the developmental dependence of the cochlea on TH is not only strict but also temporally circumscribed, and that preventing irreversible hearing loss in CH requires diagnosis and hormone replacement no later than the second week of life.

In light of the evidence reviewed, we suggest that assessment of TH signaling levels may be considered in the diagnostic workup for selected patients with idiopathic hearing loss, particularly in congenital or early-onset cases, or when clinical features suggest possible thyroid dysfunction. For patients whose HL is attributable to a TH signaling deficiency, expediting diagnosis to allow prompt initiation of thyroxine replacement therapy is advantageous, underscoring the importance of early detection. For patients with hearing loss attributable to resistance to thyroid hormone (RTH) syndrome, longitudinal data demonstrate that such deficits can worsen with age, highlighting that TH signaling impairments, even when present from birth, may confer not only developmental but also degenerative auditory risks, thereby mandating lifelong audiologic surveillance in this population [42].

While significant progress has been made, further research is needed to fully elucidate the downstream transcriptional networks and cell-specific effector mechanisms by which TH orchestrates cochlear maturation, and to explore potential therapeutic strategies targeting the local cochlear TH system beyond systemic replacement. Understanding the multifaceted role of TH in cochlear development provides crucial insights not only into the etiology of thyroid-related deafness but also into the fundamental principles governing sensory organogenesis and the vulnerability of complex developmental processes to endocrine disruption.

## Data Availability

No new data were created or analyzed in this study. Data sharing is not applicable to this article.

## Supplementary Materials

The following supporting information can be downloaded at: https://www.mdpi.com/article/10.3390/ijms27052196/s1.

## Abbreviations

ATP	Adenosine Triphosphate
CH	Congenital Hypothyroidism
DIO2	Type 2 Iodothyronine Deiodinase
DIO3	Type 3 Iodothyronine Deiodinase
EP	Endocochlear Potential
FT3	Free Triiodothyronine
FT4	Free Thyroxine
GER	Greater Epithelial Ridge
HC	Hair Cell
HL	Hearing Loss
IHC	Inner Hair Cell
IPC	Inner Pillar Cell
LER	Lesser Epithelial Ridge
MET	Mechanoelectrical Transduction
MCT8	Monocarboxylate Transporter 8
OC	Organ of Corti
OHC	Outer Hair Cell
OPC	Outer Pillar Cell
P0–P21	Postnatal Day 0–21
PC	Pillar Cell
PTU	Propylthiouracil
RTH	Resistance to Thyroid Hormone
rT3	Reverse Triiodothyronine
RXR	Retinoid X Receptor
SGN	Spiral Ganglion Neuron
SSNHL	Sudden Sensorineural Hearing Loss
SV	Stria Vascularis
T2	3,3′-Diiodothyronine
T3	Triiodothyronine
T4	Thyroxine
TC	Tunnel of Corti
TH	Thyroid Hormone
THR	Thyroid Hormone Receptor
THRα	Thyroid Hormone Receptor Alpha
THRβ	Thyroid Hormone Receptor Beta
TM	Tectorial Membrane
TR	Thyroid Hormone Receptor
TRE	Thyroid Hormone Response Element
TSH	Thyroid-Stimulating Hormone
WT	Wild-Type
