# Supplementary material for: The Role of Thyroid Hormone Signaling in the Development and Pathophysiology of Hearing: From Molecular Mechanisms to Clinical Applications
Source: Int J Mol Sci. 2026 Feb 26;27(5):2196. doi: 10.3390/ijms27052196 (PMC12984108; doi:10.3390/ijms27052196)
Supplement: Supplementary file 1 [file ijms-27-02196-s001.zip › ijms-4087566-supplementary.pdf]

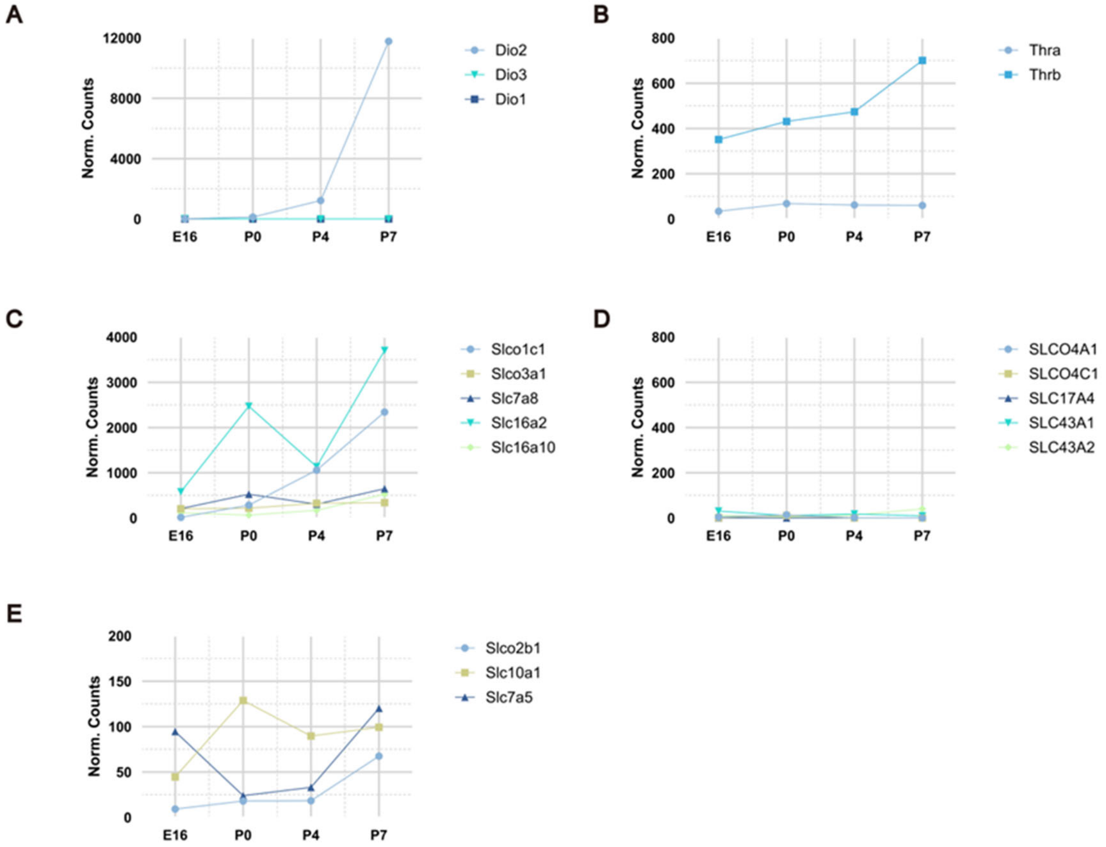

Supplementary Figure S1.

Publicly available datasets (originated from the GSE60019 dataset) in the Gene Expression SHIELD (Shared Harvard Inner-Ear Laboratory Database) were utilized for statistical analysis and the evaluation of DIO (A), THR (B), and TH transporter (C) RNA expression levels in the mice cochlea at E16, P0, P4, and P7, respectively.
